# Current and Future Trends in Magnetic Resonance Imaging Assessments of the Response of Breast Tumors to Neoadjuvant Chemotherapy

**DOI:** 10.1155/2010/919620

**Published:** 2010-09-29

**Authors:** Lori R. Arlinghaus, Xia Li, Mia Levy, David Smith, E. Brian Welch, John C. Gore, Thomas E. Yankeelov

**Affiliations:** ^1^Department of Radiology and Radiological Sciences, Institute of Imaging Science, Nashville, TN 37232-2310, USA; ^2^Department of Biomedical Informatics, Institute of Imaging Science, Nashville, TN 37232-2310, USA; ^3^Department of Medicine, Institute of Imaging Science, Nashville, TN 37232-2310, USA; ^4^Department of Biomedical Engineering, Institute of Imaging Science, Nashville, TN 37232-2310, USA; ^5^Department of Physics and Astronomy, Institute of Imaging Science, Nashville, TN 37232-2310, USA; ^6^Department of Molecular Physiology and Biophysics, Institute of Imaging Science, Nashville, TN 37232-2310, USA; ^7^Department of Cancer Biology, Institute of Imaging Science, Nashville, TN 37232-2310, USA

## Abstract

The current state-of-the-art assessment of treatment response in breast cancer is based on the response evaluation criteria in solid tumors (RECIST). RECIST reports on changes in gross morphology and divides response into one of four categories. In this paper we highlight how dynamic contrast-enhanced magnetic resonance imaging (DCE-MRI) and diffusion-weighted MRI (DW-MRI) may be able to offer earlier, and more precise, information on treatment response in the neoadjuvant setting than RECIST. We then describe how longitudinal registration of breast images and the incorporation of intelligent bioinformatics approaches with imaging data have the potential to increase the sensitivity of assessing treatment response. We conclude with a discussion of the potential benefits of breast MRI at the higher field strength of 3T. For each of these areas, we provide a review, illustrative examples from clinical trials, and offer insights into future research directions.

## 1. Introduction

In recent years, neoadjuvant chemotherapy has become increasingly important for patients with operable breast cancer requiring chemotherapy [1, 2]. There are several important reasons for this trend. These include the potential ability to reduce primary tumor burden and thereby allowing for higher rate of breast conservation surgery, as well as earlier treatment of micrometastatic disease. Neoadjuvant chemotherapy also provides an opportunity to evaluate (*in vivo*) the response of a particular treatment on an individual patient basis which may inform the selection of postoperative chemotherapy. Furthermore, according to National Surgical Adjuvant Breast and Bowel Project B-18 (NSABP B-18) results, neoadjuvant chemotherapy yields a survival outcome similar to adjuvant treatment in early breast cancer patient [3]. Thus, noninvasive imaging techniques which can report on the status of the response of breast tumors to neoadjuvant chemotherapy is of great import.

While X-ray mammography and ultrasound imaging play a critical role in the detection and diagnosis of breast cancer, they do not provide quantitative information on their response to therapy. Additionally, the 2D nature of planar X-ray mammography makes it difficult to interpret potentially complex 3D tumor structures, thus severely limiting its value in quantitative longitudinal studies. Consequently, there are currently no radiological methods for adequately assessing the state of breast tumors or their response to treatments to guide clinical decisions. Although serial biopsies may be obtained to measure changes at the cellular and molecular levels, the spatial sampling may be poor and the results misleading [4–7]. Clinical judgments using physical examination, mammograms and ultrasound to measure short-term treatment effects have a high inter-observer variability and are prone to error. The development of appropriate methods of tissue characterization that could be applied early in the course of treatment to assess response would allow clinicians to individualize therapy based on each patient's response to a particular agent. Quantitative imaging techniques which can reliably assess tumor response are urgently needed to improve clinical breast cancer care.

Three generations of solid tumor response criteria have been applied to assess treatment response in breast cancer clinical trials. The original WHO criteria developed in 1981 used the sum of two-dimensional measurements of cancer lesions to compute a composite estimate of tumor burden while the currently accepted standard method Response Evaluation Criteria in Solid Tumors (RECIST) uses single dimensional measurements as measured by ultrasound, X-ray-computed tomography (CT), or magnetic resonance imaging (MRI). While details of the criteria are presented elsewhere [8], we briefly summarize the salient features here. 

In the pretreatment scan, “target lesions are identified” and their longest dimension and perpendicular short axis are measured. For lymph node lesions, the short axis is used for the composite tumor burden score while the long axis is used for nonlymph node lesions. The “baseline sum diameters” are then defined by adding the single dimension measurement of all the target lesions. Additional scans are performed after treatment and analyzed similarly. The percent change in the sum of diameters from the baseline assessment to the follow-up assessment after treatment is calculated and this quantity is used to classify response. A complete response (CR) is defined as the disappearance of all target lesions; a partial response (PR) is defined as a 30% decrease in the sum of diameters of the target lesions; progressive disease (PD) is defined as a 20% increase in the sum of diameters of the target lesions or the appearance of any new lesions; and stable disease (SD) is defined as changes that do not meet the previous three criteria. While many clinical trials have used these criteria for assessing cancers at many disease sites (see [9] and references cited therein), it is well recognized that this approach needs to be significantly improved for a number of reasons including practical, technical, and scientific issues. Practical and technical issues included how to select the total number of lesions to include in the sum longest dimension calculation, and how to apply RECIST in trials where progression, and not response, is the primary endpoint. However, more fundamental (scientific) issues have also been raised, including whether or not to include emerging imaging techniques such as, fluorodeoxyglucose PET (FDG-PET) or dynamic contrast-enhanced MRI (DCE-MRI) with RECIST. A second fundamental issue is whether RECIST should be included—or if it is even relevant—in assessing the activities of noncytotoxic anticancer drugs. The metric for positive response is based only on anatomical and morphological changes which are (temporally) downstream manifestations of underlying pathophysiological changes which may occur earlier. The current (and presumably many future) generations of anticancer therapy use molecularly targeted agents, so changes in morphology may not be the most appropriate method to assess response.

Response criteria continue to evolve and require validation as new technologies for assessing disease emerge. In early 2009, RECIST was updated, as RECIST 1.1, and made substantial progress towards addressing these (and other) issues [9]. A major development was the incorporation of fluorodeoxyglucose positron emission tomography (FDG-PET) in the guidelines, albeit in the limited role of assessing progressive disease. This is at once exciting and sobering; exciting in the sense that this is the first time the working group has incorporated a nonanatomical imaging modality in the assessment of tumor response, a trend that will almost certainly continue in the future. However, it is also sobering because FDG-PET was first employed in a human imaging study in 1976 [10] and required 33 years to advance to the point that it could be recommended as a method for assessing one aspect (i.e., progressive disease) of tumor response. In part, this is due to the difficulty in developing, optimizing, and standardizing advanced imaging techniques to permit results to be obtained and compared across vendors and sites. In light of this, the RECIST working group concluded that “there is not sufficient standardization or evidence to abandon anatomical assessment of tumor burden”. Nonetheless, it makes clear that more advanced and specific imaging methods will be incorporated into RECIST in future years and this fact is not lost on the PET community who has begun to formulate the PET Response Criteria in Solid Tumors, or “PERCIST” [11].

Clearly, methods are needed to characterize the underlying pathophysiological changes induced by specific targeting agents. Such methods may be considerably more likely to offer earlier—and more specific—information on response to treatment when compared to changes in longest tumor dimensions. This may be especially true in the case of neoadjuvant treatment of breast cancer. Neoadjuvant treatment offers a unique opportunity for correlation of image-based changes in tumor burden with the pathologic response observed at the time of definitive surgery. Pathologic complete response is highly predictive of survival in breast cancer and offers an earlier endpoint for preliminary validation of image-based response criteria. Several aspects of MRI data analysis, acquisition, and synthesis have matured to the point where they can offer quantitative information on breast tumor status and response to therapy. 

In this contribution, we begin by defining what is meant by “quantitative imaging” and then proceed to review both diffusion weighted MRI (DW-MRI) and dynamic contrast-enhanced MRI (DCE-MRI). We then proceed to discuss how these techniques can best be synthesized (through image registration and the incorporation of bioinformatic approaches) to offer optimal insight into treatment effects. In each section we introduce the basis of the technique, offer several illustrative examples, and then highlight areas for further research. We conclude with a discussion of what high magnetic field scanners (3T), which are gradually permeating clinical practice, can offer for the quantitative imaging of breast cancer.

## 2. Beyond Signal Intensity: QuantitativeImaging

Any digital image, regardless of whether it was acquired with a common camera or a sophisticated medical imaging device, consists of a matrix of signal intensities. In the case of a photograph captured with a camera, the signal intensities in each pixel are given by a combination of the colors red, green, and blue, whereas in a medical image, the displayed signal intensity can be made proportional to one or more physical or physiological properties of tissue. In standard of care clinical imaging, the signal intensities in each voxel are acquired in such a way so as to optimize the contrasts between different tissue types. This improves the ability of a clinician to detect abnormalities and allows for investigators to apply the RECIST criteria described above. However, the signal intensities are usually a complicated mixture of several different quantities related to both physiology and scanner characteristics that do not have physical units associated with them.

Quantitative imaging connotes techniques in which the signal intensity in each voxel does have physical units and is therefore associated with a particular physical or physiological phenomenon. For example, there are techniques available that report on tissue oxygen status, blood flow, or metabolism; in each of these cases, the signal intensities within the image can be made proportional to mmHg, mL(blood)/mL(tissue)/min, and the rate of glucose metabolism, respectively. Quantitative cancer imaging is motivated by the hypothesis that changes in morphology are only the downstream manifestations of underlying pathophysiological changes (such as those just above), but that if it were possible to reliably measure more relevant properties we would be able to assess treatment response at an early stage and ultimately, perhaps, contribute to individualized patient care. Given the fact that anticancer treatments are increasingly specific, it is necessary to develop diagnostic techniques that can report on the specific effects of those treatments.

## 3. DCE-MRI

### 3.1. Fundamentals of DCE-MRI

DCE-MRI involves the rapid acquisition of images before, during, and after the injection of a contrast agent (CA). MRI CAs are pharmaceuticals administered to patients that are designed to increase the contrast between different tissues by changing a tissue's inherent relaxation times, *T*_1_ and/or *T_2_*. These relaxation properties reflect the time it takes for the nuclear magnetization, induced by the external polarizing magnetic field, to recover back towards equilibrium after being disturbed by radiofrequency emissions during the MRI process. The most common MRI CAs are gadolinium-chelates such as, Gd-DTPA. In a typical DCE-MRI imaging session, a region of interest (ROI) is selected for study (e.g., a tumor locus), and a series of heavily *T*_1_-weighted MR images are collected before, during, and after a CA is injected into the antecubital vein of a patient. Each image in the series corresponds to one time point, and each pixel in each image set gives rise to its own signal intensity time course which can be analyzed mathematically. In general, quantitative analyses are based on compartmental modeling whereby the tissue within the tissue element corresponding to each voxel is divided into a series of partitions, between which contrast agent may move back and forth. In the case of DCE-MRI, each voxel is typically assumed to consist of two compartments, the vascular space and the extravascular space. By considering the rate of change of the concentration of CA in extravascular space, we can write down the following model:

(1) $$\frac{d}{dt}C_{t}\left(t\right)=k_{1}C_{p}\left(t\right)-k_{2}C_{t}\left(t\right),$$

where *C_t_*(*t*) and *C_p_*(*t*) are the time course of concentrations of CA in the tissue and plasma space, respectively, and *k*_1_ and *k_2_* are the rate constants describing the exchange of the CA from plasma to tissue and tissue to plasma, respectively. By convention, *K* ^trans^ and *v_e_* denote the volume transfer constant and the volume of extravascular extracellular space, respectively. It can readily be shown [12] that *k*_1_ = *K* ^trans^ and *k_2_* = *K* ^trans^/*v_e_*. With these assignments, (1) (details provided in [12]) has the following solution:

(2) $$C_{t}\left(T\right)=K^{\text{trans}}\overset{T}{\underset{0}{\int }}C_{p}\left(t\right)\exp\;\left(-\frac{K^{\text{trans}}}{v_{e}\left(t-T\right)}\right)dt.$$

Equation (2) is frequently referred to as the standard model and can be extended to include a vascularcomponent explicitly in which case the model is given by (3)

(3) $$C_{t}\left(T\right)=K^{\text{trans}}\overset{T}{\underset{0}{\int }}C_{p}\left(t\right)\exp\;\left(-\frac{K^{\text{trans}}}{v_{e}\left(t-T\right)}\right)dt+v_{p}C_{p}\left(T\right),$$

where *v_p_* is the plasma volume fraction. In situations where the blood plasma volume is very small (say, less than 2%), (2) may be adequate to assess the DCE data. However, as *v_p_* gets larger, (2) becomes less applicable and (3) is preferred. Of course, *a priori*, the approximate plasma volume of the tumor may not be known and that is why many investigators prefer using (3) to fit DCE time courses.

The potential clinical utility of *K* ^trans^ and *v_p_* is that they offer the ability to noninvasively and quantitatively report on tumor vascular status; *K* ^trans^ estimates tumor vessel blood flow and permeable, while *v_p_* reports on the volume fraction of a section of tissue that consists of blood. Both of these measures can be used in studies wherein the therapy is designed to affect tumor vascular status. Changes in the extravascular extracellular volume fraction (as estimated by *v_e_*) are more complicated to interpret as there are many treatments that might change the volume and geometry of this space. Most studies that employ DCE-MRI to assess treatment response focus on *K* ^trans^ and *v_p_*_._

**Figure 1:**
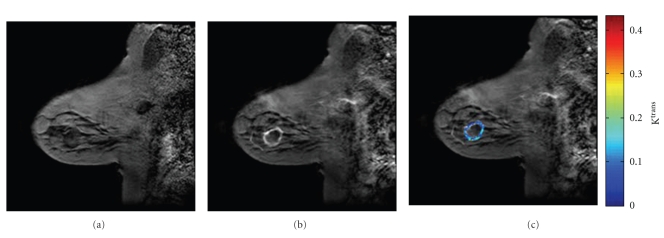
(a) and (b) display pre-contrast and postcontrast *T*_1_-weighted sagittal images of a breast tumor. By considering the time course from each voxel, a parametric map can be generated that reports on, for example, the volume transfer constant (*K* ^trans^) as displayed in (c).

In order to perform quantitative DCE-MRI analysis, the time courses of *C_t_*(*t*) and *C_p_*(*t*) must be estimated, then those time courses can be input into a curve-fitting routine to extract *K* ^trans^, *v_p_*, and *v_e_* on a voxel-by-voxel or region-of-interest basis. Figure 1 presents an example of this approach. In DCE-MRI this is not a straightforward process because the CA is not directly measured—only its affect on the tissue's native *T*_1_ relaxation is measured in the study. Thus, the MR signal time course must be converted to a time course representing the concentration of CA. The majority of studies employ (4) for this transformation:

(4) $$R_{1}=r_{1}C_{x}+R_{10},$$

where *R*_1_ = 1/*T*_1_, *r*_1_ is the relaxivity of the CA (which measures the efficacy of the CA at enhancing relaxation in units of mM^−1^s^−1^), *C_x_* is *C_t_* or *C_p_*, and *R_10_* = 1/*T_10_*, where *T_10_* is the longitudinal relaxation time prior to administration of CA. Equation (4) assumes that the extravascular space is a homogeneous solution and that the system remains in what is called the “fast exchange limit” (FXL) with respect to the water exchange between the extravascular extracellular space and the extravascular intracellular space. In most tissues, most water is intracellular, and because the common Gadolinium chelates cannot access this intracellular water directly, water exchange between the extravascular extracellular space and the extravascular intracellular space must be explicitly incorporated into analytic models under certain circumstances [13, 14]. Similar comments apply to water exchange between the intravascular and extravascular spaces when using an intravascular agent [15]. In these cases, longitudinal relaxation may exhibit a recovery that is not well described by a single exponential or single time constant as discussed by several investigators [16–18]. The effects of slower water exchange may be incorporated into analyses of the MRI signal and then the longitudinal relaxation can often be characterized as exhibiting a bi-exponential decay. More specifically, the signal intensity equation in a spoiled gradient recalled echo acquisition (the main choice for DCE-MRI studies) is given by

(5) $$\frac{S}{S_{0}}=\frac{1-\exp\;\left(-TR/T_{1}\right)\cdot \sin\;\alpha }{1-\cos\;\alpha \cdot \exp\;\left(-TR/T_{1}\right)},$$

where *α* is the flip angle, *S*_0_ is a reference signal that depends on the scanner gain and proton density, *TR* is the repetition time, and we have assumed that the echo time (*TE*) is much less than the apparent transverse relaxation rate T_2_*. However, in the case of bi-exponential decay of longitudinal relaxation, (5) must be amended to

(6) $$\begin{array}{cc} \frac{S}{S_{0}} & =a_{L}\frac{1-\exp\;\left(-TR/{T_{1}}_{L}\right)\cdot \sin\;\alpha }{1-\cos\;\alpha \cdot \exp\;\left(-TR/T_{1L}\right)}\\ & \;+a_{S}\frac{1-\exp\;\left(-TR/{T_{1}}_{S}\right)\cdot \sin\;\alpha }{1-\cos\;\alpha \cdot \exp\;\left(-TR/T_{1S}\right)}, \end{array}$$

where *a_L_* and *R_1L_* are the fraction and relaxation rates, respectively, for the component with the longer *T*_1_ (i.e., *R_1L_* *≡* 1/*T_1L_*); *a_S_* and *R_1S_* are defined similarly for the component with the shorter *T*_1_. Furthermore, instead of (4) we have

(7) $$\begin{array}{cc} & \frac{1}{T_{1S,1L}}\\ & \;\;=\frac{1}{2}\left(\frac{1}{T_{1A}}+\frac{1}{\tau_{A}}+\frac{1}{T_{1B}}+\frac{1}{\tau_{B}}\right)\\ & \;\;\;\pm \;\frac{1}{2}[{\left(\frac{1}{T_{1A}}+\frac{1}{\tau_{A}}+\frac{1}{T_{1B}}+\frac{1}{\tau_{B}}\right)}^{2}\;\\ & \;\;\;{\;\;\;-\left(\left(\frac{1}{T_{1A}}+\frac{1}{\tau_{A}}\right)\left(\frac{1}{T_{1B}}-\frac{1}{\tau_{B}}\right)-\frac{1}{\tau_{A}\tau_{B}}\right)]}^{1/2}, \end{array}$$

where *T*_1*A*_ and *T*_1*B*_ are the relaxation times for components *A* and *B*, respectively, *τ*_*A*_ denotes the average lifetime of a water proton in compartment *A*, and *τ*_*B*_ denotes the average lifetime in the compartment *B*. In this formalism the extravascular extracellular space can be taken to be compartment *A* while the extravascular intracellular space can be taken to be compartment *B*. The pharmacokinetic parameters obtained using (2), (4), and (5) (or (3), (4), and (5)) and those extracted by (2), (6), and (7) may differ significantly [14, 19–21] and these differences have a significant effect on establishing if a tumor is responding to treatment. However, there is some disagreement as to whether this formalism is truly justified in a standard DCE-MRI study and it is therefore an active area of investigation [22].

The various models have their respective strengths and weaknesses; the FXL models with and without a *v_p_* term are straightforward to apply, but may not be as physically accurate as the exchange models. However, the exchange models require specialized data (high temporal resolution and high signal-to-noise data) that may not always be available in the clinical setting. Determining the optimal DCE analysis method for breast studies is an area of active investigation.

### 3.2. DCE-MRI in Breast Cancer Clinical Trials

A considerable number of studies of cancer have applied DCE-MRI methods but have employed only qualitative or semi-quantitative analyses of these data, that is, the investigators did not perform complete modeling, rather they studied features such as, the volume of the enhancing region and the area under the signal intensity time course, and so forth. There have been substantially less studies that have included quantitative modeling of DCE-MRI time courses (see, e.g., [23–25]), and when quantitative models have been employed, different authors have not always shown similar results.

To date, the most complete study of contrast-enhanced MRI in assessing the response of breast tumors to therapy was reported by Ah-See et al. [26] who applied both DCE-MRI and dynamic susceptibility MRI (DSC-MRI, for a review see [27]). The authors studied the ability of *K* ^trans^, *k*_ep_ (*≡* *K* ^trans^/*v_e_*), *v_e_*, maximum contrast agent concentration, relative blood flow, relative blood volume, and mean transit time to predict treatment response in 28 patients receiving 5-fluorouracil, epirubicin and cyclophosphamide neoadjuvant chemotherapy. While all parameters correlated with pathological response, *K* ^trans^ was the best predictor with an area under the receiver operating characteristic curve of 0.93 while changes in tumor size did not predict for pathologic response. This is a notable result because it offers evidence of an emerging functional imaging technique that outperforms conventional morphological imaging.

However, a similar study by Yu et al. reported somewhat different results [28]. These investigators studied the ability of tumor size, *K* ^trans^ and *k*_ep_ to predict final clinical response based on changes after two and four cycles of anthracycline and cyclophosphamide neoadjuvant chemotherapy in 29 patients with invasive breast cancer. While there was a significant correlation between tumor size and the pharmacokinetic parameters, the area under the receiver operating characteristic curve differentiating between nonresponders and responders was 0.88, 0.77, and 0.63 for tumor size,*k*_ep_,and *K* ^trans^, respectively. The authors concluded that early tumor size changes on MRI after one cycle of therapy provided the most accurate predictor in assessing response to this treatment regimen. 

The reasons for such discrepancies are not entirely clear but almost certainly related to the differences in treatment regimen and data acquisition techniques and timing of the second imaging session. (The two groups actually used very similar analysis techniques based on (2) described above.) In particular, Ah-see et al. acquired the second imaging data set after the second cycle of therapy, while Yu et al. acquired the second imaging data set after the first cycle of therapy. It is possible that the changes observed by the two groups are due to the timing of the second imaging session. Also, of particular interest is the issue of temporal resolution and contrast agent injection rate. Ah-See et al. acquired data with a temporal resolution of 12 seconds during their dynamic study after injection of 0.1 mmol/kg of Magnevist (Bayer Pharmaceuticals) at 4 mL/sec, whereas Yu et al. acquired data with a 42-second temporal resolution after the injection of 0.1 mmol/kg of Omniscan (GE Healthcare) at the rate of approximately 1 mL/sec. (As we will see below, the issue of temporal versus spatial resolution is of critical importance and is a difficult balancing act.) The accuracy of estimating *K* ^trans^ is determined by the initial rapid changes of the signal intensity time course so if the curve is not sampled adequately errors in *K* ^trans^ may result [29]. It is possible that the differences in temporal resolutions and injection rates led to the differences in *K* ^trans^ estimates, and it is generally appreciated that differences in acquisition protocol can lead to substantially different outcomes [30]. 

### 3.3. Research Opportunities in DCE-MRI of the Breast

As described in Section 3.1, there are a number of methods available for both data acquisition and analysis in a DCE-MRI study and the optimal methods have yet to be identified. For DCE-MRI to have the broadest impact, it will be necessary to establish the most accurate and precise methods for assessing treatment response. It is imperative, particulary when performing multi-institutional studies, to have a standard technique in place for which reproducibility at each site has been established. A particular aspect of the analysis that must be addressed is that of the arterial input function.

Before a curve fitting routine can be employed to extract the pharmacokinetic parameters discussed above, the time rate of change of the concentration of CA in the blood plasma (*C_p_*), which is typically called the arterial input function (AIF), must be established. This time course changes very rapidly, so images have to be acquired with high temporal resolution, but when images are acquired quickly there is a net decrease in signal-to-noise ratio and/or spatial resolution. The need to acquire high temporal resolution data confines quantitative DCE-MRI in two fundamental ways. The first limitation is that it limits the ability to probe tumor heterogeneity and scan large sections of tissue at high resolution. The second limitation is that high temporal resolution acquisitions limit the clinical adoption of the technique because high spatial resolution data covering large sections of tissue are required for clinical reading and applying the RECIST criteria; in particular, high temporal resolution data is usually limited to scanning one breast which contrasts with the clinical aim of examining both breasts. Some investigators have attempted to overcome this problem by using a population-based AIF [31, 32], reference region models [33, 34], or keyhole imaging techniques [35, 36]. Currently, there are three major kinds of AIFs employed in breast studies, in increasing order of difficulty to obtain, these are: (1) a bi-exponential decay model; (2) a population based AIF, or (3) an individually measured AIF. The first of these does not require any new data acquisition or analysis, while the second method requires measurement of the AIF in a suitable population of patients or volunteers. The third method is the most complex but also (potentially) the most accurate. Using a model or population based AIF reduces the demands on the data acquired, but if there is a substantial difference between the model or population AIF and an individual's AIF then this may lead to significant errors in the pharmacokinetic parameters returned from such an analysis. The accurate characterization of the AIF represents an aspect of breast DCE-MRI that could benefit from further research.

## 4. DW-MRI

### 4.1. Fundamentals of DW-MRI

The contrast mechanism behind DW-MRI is based upon the effects of tissue microstructures and organization on the self-diffusion of water. The random motions of water molecules, also known as Brownian motion, are due to thermal energy and were first analyzed mathematically by Einstein [37]. For freely diffusing molecules, the distribution of displacements that the particles will experience over a given amount of time (*t*) is described by a Gaussian function. The standard deviation is determined by the distribution which is defined by the diffusion coefficient (*D*) of the molecules, which depends upon the temperature, the size of the particle, and the viscosity of the medium. The mean-squared displacement of the molecules in one dimension over the diffusion time *t* is given by the equation

(8) $$\langle x^{2}\rangle =2Dt.$$

For example, free diffusion of water molecules at normal body temperature, with a diffusion coefficient of 3×10^−3^ mm^2^/s, would result in an average displacement of 17–25 *μ*m in one dimension over diffusion times of 50–100 ms. However, water molecules within tissue are not freely diffusing on this time scale. They encounter macromolecules, cell membranes, and other tissue microstructures that hinder diffusion, causing a reduction in the average diffusion displacement, compared to that of free water. This effective, or apparent diffusion coefficient (ADC) can be measured with DW-MRI [38].

The signal intensity for each voxel acquired in DW-MRI is dependent on the apparent diffusion coefficient of water (we now use the term *D* interchangeably with *ADC* in a single direction), the signal acquired with no diffusion weighting (*S*_0_), and a diffusion-weighting factor, *b *[39]:

(9) $$S=S_{0}\exp\;\left(-bD\right),$$

where

(10) $$b=\gamma^{2}\delta^{2}G^{2}\left(\Delta -\frac{\delta }{3}\right),$$

*γ* is the gyromagnetic ratio of protons, *δ* is the duration of the diffusion-weighting gradient G, and Δ is the time (equivalent to t in (8)) over which diffusion effects on the MRI signal are manifest.

The value of *b* is known and determined by the imaging parameters. Therefore, only two images, one nondiffusion-weighted image (*G* = 0) and one diffusion-weighted image (*G* > 0) are needed to obtain *S*_0_ and *S*, respectively, and to calculate a map of *D* in a single direction. In practice, however, it is common to apply diffusion-weighting in three orthogonal directions (i.e., *x*, *y*, and *z*) where *G_x_* = *G_y_* = *G_z_* > 0. The ADC maps calculated from the three images are then averaged to obtain a mean ADC map, reducing the effects of anisotropic diffusion.

### 4.2. DW-MRI in Breast Cancer Clinical Trials

**Figure 2:**
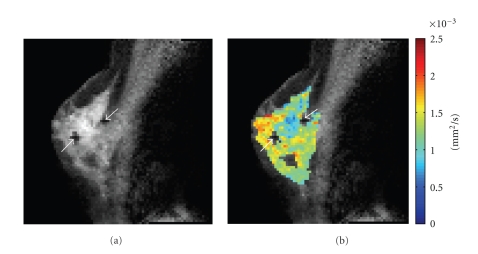
ADC values are reduced in breast cancer tumors compared to the unaffected tissue. Shown here is a postcontrast *T*_1_-weighted image (a) for reference and an overlay of the corresponding ADC map (b). The tumor, located above and between the biopsy clips (white arrows), exhibits increased signal in the *T*_1_-weighted image and decreased ADC values, compared to the surrounding tissue.

Tumors typically exhibit decreased ADC values compared to the surrounding unaffected tissue because of their increased cellularity [40–42] (e.g., see Figure 2). The sensitivity of DW-MRI to these changes in tissue microstructure and organization has led to increasing interest in the potential of ADC measurements as a biomarker for diagnosis of cancer, tumor differentiation, and treatment assessment. Preclinical studies of the effects of successful treatment on tumor ADC values produced provocative results. For example, Galons et al. [43] demonstrated that successful treatment of human breast cancer xenografts with Paclitaxel resulted in a significant increase in tumor ADC within just two days of the initiation of treatment, and these changes preceded significant reductions in tumor volume. 

Although there have been few clinical studies of the effects of treatment on tumor ADC in breast cancer published to date, the results of the existing studies are promising. In a study of 11 women with biopsy-proven infiltrating breast cancer undergoing neoadjuvant chemotherapy (Taxotere or adriamycin/Cytoxan), Yankeelov et al. reported that the mean tumor ADC value increased significantly (*P* = .005) by 24% after a full course of chemotherapy [44]. While this study suggests that ADC may be a useful imaging biomarker for assessing overall response to therapy in breast tumors, it does not provide insight into its usefulness as an early biomarker. Evidence of early changes in ADC due to treatment was reported by Pickles et al. [45]. In this study, both tumor longest diameter and ADC values were measured in eight women with biopsy-proven invasive breast cancer who were undergoing neoadjuvant chemotherapy (four cycles of epirubicin and cyclophosphamide). Measurements were made prior to the start, after the first cycle, and after the second cycle of therapy. A significant increase (14%, *P* = .005) in mean tumor ADC was observed after a single cycle of chemotherapy, while tumor longest diameter remained relatively unchanged (−1%, *P* = .852). After two treatment cycles, the tumor longest diameter began to approach a significant decrease (−20%, *P* = .057) compared to the pretreatment value, and the mean ADC value was 27% higher than the pretreatment value (*P* = .004). 

More recently, Sharma et al. [46] compared tumor ADC values at multiple time points throughout the course of therapy in a study of 56 women with cytologically proven infiltrating ductal carcinoma undergoing neoadjuvant chemotherapy (cyclophosphamide, adriamycin (doxorubicin) and 5-fluorouracil (5-FU); cyclophosphamide, epirubicin and 5-FU; or paclitaxel and epirubicid). ADC measurements were made before, after the first cycle, after the second cycle, and after the third cycle of treatment, although not all participants were able to be scanned at all four time points. Overall, the mean tumor ADC increased significantly (*P* < .01) from 0.95 ± 0.11 × 10^−3^ mm^2^/s (*N* = 53) before treatment to 1.09 ± 0.16 × 10^−3^ mm^2^/s (*N* = 14) after 1 treatment cycle. In the 11 patients scanned both before treatment and after one treatment cycle, tumor ADC increased significantly (*P* = .002) from 1.01 ± 0.07 × 10^−3^ mm^2^/s before treatment to 1.15 ± 0.13 × 10^−3^ mm^2^/s. Mean tumor ADC continued to increase significantly (*P* < .01) throughout treatment to 1.21 ± 0.22 × 10^−3^ mm^2^/s (*N* = 24) after the second treatment cycle and 1.30 ± 0.24 × 10^−3^ mm^2^/s (*N* = 29) after the third treatment cycle. Tumor diameter and volume reductions were observed only after the second cycle of treatment. The authors suggest that changes in ADC may be more useful for personalized treatment management than the current standard of changes in tumor morphology.

### 4.3. Research Opportunities in DW-MRI of the Breast

While the results of the DW-MRI studies mentioned in the previous section are encouraging, the application of DW-MRI in breast cancer is still relatively new and there are several technical challenges that need to be addressed. First, the chemotherapy agents used in these studies were all cytotoxic. Successful treatment with these agents leads to cell death, ultimately increasing the extracellular volume and/or reducing the cell density within the tumor, which would be expected to lead to an increase in the tumor ADC. The effects of hormonal treatments and antiangiogenic and cytostatic chemotherapy agents on the tumor ADC are not fully understood yet and need to be explored. 

Timing of the ADC measurements is another issue that has not been fully addressed yet. In preclinical studies, a significant increase in ADC in response to treatment has been measured in as few as two days [43]. However, the earliest posttreatment measurements in the clinical studies mentioned above were acquired after the first course of treatment, typically three to four weeks after the start of treatment [45, 46]. The course of tumor ADC changes over time is not fully understood. For example, tumor ADC values may initially increase as tumor cells die and then decrease as healthy tissue replaces it. 

Cardiac, respiratory, and bulk subject motion during diffusion-encoding can introduce artifacts into the images and lead to inaccurate ADC measurements. To avoid this, a fast imaging technique called echo planar imaging (EPI) [47] is typically employed. Unfortunately, EPI is prone to severe image distortions at the interface of materials with different magnetic susceptibilities, such as, the air/tissue and adipose/glandular tissue interfaces in the breast and chest wall. Alternative fast image acquisition schemes, such as fast spin echo (FSE) [48], which are not as susceptible to these distortions can be used to prevent them. However, these alternative acquisition schemes are more prone to motion-induced artifacts and often require the collection of additional images to correct the motion-induced artifacts. Susceptibility-induced distortions can be corrected using image post-processing techniques; however, this requires the acquisition of an accurate map of the main magnetic field [49]. 

Subject motion between the acquisition of slices or different diffusion-weightings can cause misregistration between the nondiffusion-weighted and diffusion-weighted images, leading to errors in the ADC calculation. Multiple repetitions are often acquired and averaged to increase signal to noise, and misregistration between the individual images will also lead to inaccurate ADC calculations. Slice-based affine registration of the individual diffusion-weighted images to the nondiffusion-weighted image is typically used in DW-MRI studies of the brain. Until recently, however, these techniques have not been used in DW-MRI studies of the breast [50].

Although guidelines have been published [51], little consensus exists yet within the field regarding the optimum set of *b*-values that should be used in DW-MRI of breast cancer. Blood flow in the capillaries causes an artificial decrease in signal at very low *b*-values (e.g., 0–100 s/mm^2^). This effect is related to the perfusion fraction, which is the fraction of total water located within the capillaries in the tissue [52]. Tumors typically exhibit a broad range of perfusion factors (20%–80% [53, 54]), increasing the effects of perfusion on the signal measured at low *b*-values. Therefore, values of 50–100 s/mm^2^ are typically used instead of *b* = 0 s/mm^2^ in cancer applications. At higher *b*-values (e.g., ≥3000 s/mm^2^), there is greater signal attenuation due to diffusion and the measured signal may appear to be artificially increased by rectified noise in magnitude images as the noise floor is reached. The rate at which the noise floor is approached is related to the ADC value; the higher the ADC value, the faster the signal is attenuated at a lower *b*-value. Therefore, the optimal high *b*-value for estimation of ADC values is related to the ADC value of the tissue being probed. A wide range of high *b*-values were used in the studies described in Section 4.2 (300–1000 s/mm^2^). The use of multiple *b*-values can mitigate this confusion but requires additional images and therefore takes more time. 

In addition to the technical challenges of DW-MRI acquisition, there exist challenges related to the analysis methods used in longitudinal studies of ADC. The most common method is region-of-interest (ROI) analysis, where ADC values are averaged over a manually defined set of voxels within the tumor. These mean ADC values are then compared between measurements. This method is straightforward and relatively easy to implement. However, it is prone to partial volume effects and inter- and intrauser errors in ROI selection. Voxelwise statistical comparisons may provide a better means of analyzing tumors, particularly if the tumors exhibit heterogeneous ADC values. Ma et al. [55] recently applied a Voxelwise analysis method called “functional diffusion mapping” (fDM) in a small study evaluating early treatment response in breast cancer. Voxel-based analyses require coregistration of images over time, and there are several challenges to doing this, which will be described in more detail in the next section.

## 5. Longitudinal Registration of Breast Images

### 5.1. Fundamentals of Image Registration

Image registration is the process of calculating a transformation to align one image to a target image. It has been widely used in the field of medical image analysis to, for example, integrate complementary information from multiple modalities, estimate the variations in anatomical structures within a population, follow diseases over time, or detect tumor response to therapy.

Two key elements are involved in image registration: calculation of a function which measures the similarity between two images such as their mutual information or a cost function that quantifies differences, and a transformation which maps corresponding points of two images. The goal of registration is to find an optimum transformation which matches two images maximally through minimizing/maximizing the cost/similarity function. The transformation is usually categorized as rigid, affine, or nonrigid. Only rotation and translation are involved in rigid body transformation, while there are additional scaling and shearing parameters in affine transformation. Nonrigid transformation is used to incorporate additional local elastic deformations. In general, nonrigid deformation fields can be modeled by a linear combination of basis functions which can be expressed in various ways [56–62], cubic B-splines [59, 60], or Wu's compactly supported positive definite radial basis functions [61, 62].

Parametric registration methods can be classified as point-based, surface-based, or intensity-based. Point-based registration algorithms require identifying and localizing fiducial points in two images. Fiducial points can be markers placed in images intentionally for easy identification or salient points in anatomical structures. The cost function could be the distance between two sets of points (e.g., for rigid body registration) or with additional constraints (e.g., a penalty term [63] to regularize the transformation in nonrigid registration). The rigid or affine transformation parameters or the coefficients of basis functions need to be calculated through optimizing the cost function during registration. 

Surface representation needs to be determined first and then registered in surface-based registration methods. The representation includes salient points, boundaries or curves of anatomical structures, or regions with homogeneous intensities. Various surface-based registration algorithms have been developed. The iterative closest point (ICP) [64] is one of the most robust methods applied widely for the rigid body registration of medical images. This algorithm minimizes the differences between two sets of points and calculates the transformation iteratively. The robust point matching (RPM) algorithm [65] is another approach commonly used in nonrigid registration tasks. Instead of generating a one-to-one correspondence between two sets of points as in ICP, RPM builds a “fuzzy” correspondence matrix whose elements are weights assigned to each point in one image, corresponding to each point in the other image. A virtual correspondence is then calculated based on the matrix and the TPS based transformation is obtained iteratively. 

Intensity-based registration methods employ the image intensity of each pixel (or voxel) to conduct the alignment, thus containing more comprehensive information than point- and surface-based registration. Among numerous cost or similarity functions, mutual information (MI) and normalized mutual information (NMI) [66–68] are the most common approaches in medical image registration [69–72]. MI measures the statistical dependence between two images, instead of using the image intensity directly. NMI is less sensitive to the overlap size between two images than MI [68]. MI or NMI has been employed widely as a successful similarity function and can be extended to solve more registration problems.

The MI-based rigid registration algorithm has been proposed to calculate rigid body transformation through maximizing MI using Powell's optimization method [71]. One of the representative MI-based nonrigid registration algorithms [73] combines a global transformation (rigid or affine) and a local transformation (the B-spline-based free form deformation) to model the deformation field. The cost function is composed of a similarity term (NMI) and a smoothness term (the bending energy [63]) and optimized using the gradient descent technique. The method was applied to breast MR images and then evaluated by a finite element method- (FEM-) based validation algorithm [74]. Another popular MI-based nonrigid registration algorithm is the adaptive bases algorithm (ABA) [61]. Wu's [62] radial basis functions are compactly supported and employed to compute the transformation. The coefficients of basis functions are searched using a gradient descent algorithm combined with a line minimization algorithm. 

In addition to parametric registration algorithms, medical images can also be aligned using nonparametric algorithms, such as the hierarchical attribute matching mechanism for elastic registration technique [75], Maxwell's demons-based registration [76], or fluid registration [77]. Those techniques compute the entire deformation field without using basis functions.

### 5.2. Review of the Literature

Although RECIST is the most widely used approach to measure tumor response, it has well-recognized deficiencies including discarding important tumor spatial information. This is also true of methods which report the mean, skewness, or kurtosis of, for example, a *K* ^trans^ distribution. The registration of longitudinal breast cancer images is an alternative approach which may allow for quantitative assessment of changes in tumor characteristics on a voxel-by-voxel basis. However, there are only a few investigations regarding the registration of longitudinal breast images, although there are a number of methods and techniques which have been developed to register a variety of imaging data. Registering breast images presents a number of difficulties that are not necessarily encountered in the registration of other organs. The challenges include bulk breast motion and deformation caused by patient repositioning, in addition to any changes in the tumor induced by therapy. The most challenging problem is that common registration techniques will compress the breast tumor imaged before treatment to match the tumor shape observed after treatment because the tumor shape typically changes and the tumor size decreases posttreatment. This kind of compression is misleading in regards to tumor response. Hence, any longitudinal breast image registration technique must be able to accommodate changes within the breast without forcing posttreatment tumor shape and volume to match pretreatment features. 

Chittineni et al. [78] presented a work which used a B-spline-based method to register longitudinal breast MR images. To maintain tumor volumes, a rigidity constraint was imposed on the nonrigid registration. Specifically, the B-spline control points that cover the tumor were forced to remain equidistant during the registration process. 

**Figure 3:**
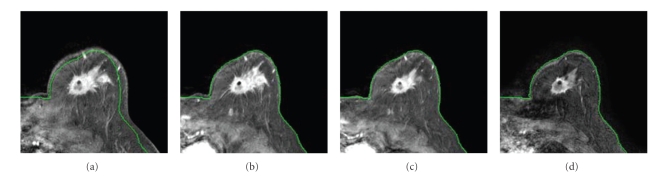
The *T*_1_-weighted MR image before neoadjuvant chemotherapy (a) is registered to the image posttreatment (d) using the constrained ABA algorithm and the original ABA algorithm, respectively. The registered image using the constrained algorithm (b) shows that the tumor is preserved successfully, while the original ABA algorithm compresses the tumor substantially (c).

Li et al. [79] proposed another registration method to align breast MR images obtained at different time points throughout the course of chemotherapy. The adaptive basis algorithm (ABA), which can accommodate nonrigid coregistrations, was extended through incorporating a volume-preserving constraint [80] into the MI-based cost function. The algorithm was used to register high-resolution *T*_1_-weighted MR images obtained pretreatment, post one cycle of treatment, and after all cycles of treatment. The generated transformations were then applied to longitudinal parametric maps obtained from breast DCE-MRI data. The alignment of the parameters which were related to tumor characteristics allowed a voxel-by-voxel-based analysis of tumor response. Li et al. [81] also developed a realistic phantom based on biological and clinical observations to quantitatively and comprehensively evaluate the proposed registration algorithm. The validation experiments demonstrated that the proposed method was highly accurate. Figure 3 presents an exampleof the performance of the algorithm. The *T*_1_-weighted MR image before neoadjuvant chemotherapy (panel a) is registered to the image posttreatment (panel d) using the constrained algorithm and the original ABA algorithm, respectively. The registered image obtained with the constrained algorithm (panel b) shows the tumor is preserved successfully, while the original ABA algorithm compresses the tumor substantially (panel c). 

As mentioned above, Ma et al. [55] conducted voxel-by-voxel analyses on ADC maps to evaluate breast cancer treatment through registering diffusion weighted MRI scans obtained pretreatment, early posttreatment, and late posttreatment. The registration algorithm [82] used by them required the user selection of control points. The TPS transformation mapping two sets of control points was calculated and applied to the images and the MI between two images was then evaluated. The MI-based cost function was minimized through automatically adjusting the locations of control points in the optimization process and recalculating the TPS transformation and the MI iteratively. The voxel-based analysis showed that ADC changes were able to predict early breast tumor response to treatment. 

Though these are exciting and promising early efforts, we stress that longitudinal registration of breast images obtained at different time points is still very much experimental and much work is left to be done.

### 5.3. Research Opportunities in Longitudinal Registration of Breast Images

The paucity of studies relating to longitudinal registration of breast images leaves many research opportunities. All current investigations rely on intensity-based registration algorithms, neglecting the inherent physical properties of tumor and healthy tissues. One potential approach is to incorporate breast tissue characteristics into the registration process. Schnabel et al. [74] constructed an FEM model which simulated biomechanical tissue deformations to validate a nonrigid registration algorithm, but the model was applied to pre- and postcontrast breast MR images, instead of pre- and posttreatment data. In addition to the challenging problems described above in longitudinal breast image registration, it is also difficult to measure tumor and tissue mechanical features on an individual basis and build patient-specific models. Furthermore, the breast tumor generally has different material properties before and after treatment. Solving those difficulties may improve the performance of registration substantially. Another potential opportunity is the use of physiological DCE-MRI parameters during registration. Integrating the parameters related to tumor features into the registration will possibly improve the performance of registration.

## 6. Biomedical Informatics Systems to Support Image-Based Treatment Response Assessment

Current approaches for applying and developing response criteria have significant limitations. In this section, we describe the current approaches and systems used to apply and develop response criteria, describe the limitations of these approaches, existing systems that may offer solutions, and additional research opportunities. 

### 6.1. Limitations of Current Approaches to Applying Response Criteria

There are several challenges with the current approach to manage clinical trial response data and apply response criteria. First, the raw imaging data are not routinely stored as part of clinical trial data management systems. The DICOM (digital imaging and communications in medicine) image files are often stored on CDs that can be shipped to central sites for review or maintained on the local institution's PACS (picture archiving and communications system). As a result, many large multicenter clinical trials often have incomplete image data sets when it is time to perform a secondary central review. Second, the current radiologist-oncologist communication paradigm is not optimally coordinated with respect to tracking target lesions of interest, resulting in incomplete information for evaluating tumor response status being recorded in the medical record [83]. Current reporting of radiology results in the medical record focuses on summarizing findings, which is insufficient for consistent application of quantitative methods to evaluate response to treatment. 

Furthermore, the use of manually transcribed records of response has several disadvantages including transcription errors, ambiguity of lesion identifiers when multiple lesions are present in the same organ or image, lack of a direct link to the source image data, and limited functionality for calculating response. Response criteria contain multiple calculation and classification rules that are often inconsistently applied within and across studies. This makes it difficult to compare trial response outcomes between cohort arms within the same study and between different trials within the same clinical domain. Automated interpretation methods are needed to improve the consistency of response classification. However, manual data collection in unstructured data formats does not facilitate automated data interpretation. The lack of a direct link between the recorded lesion values and the raw image data source also makes it difficult for outside reviewers to audit the response assessment interpretation results. Finally, there is often a delay in formal application of response criteria to assist treatment decision making and the response data are not integrated into the electronic medical record in such a way that it can readily be used at the point of care by the treating oncologists. Clinical and clinical research systems are needed to (1) support the transfer, storage, and retrieval of clinical and clinical research DICOM files, (2) manage the workflow for interpretation of image data, (3) acquire, store, and retrieve image interpretation meta-data, (4) interpret image findings for response assessment, and (5) enable review of image meta-data and their interpretations with other clinical data as needed for treatment decision making and the aggregation and auditing of response outcomes in clinical trials.

### 6.2. Limitations of Current Approaches to Development of Response Criteria

Several European and American research centers are involved in the development and testing of new oncology response assessment criteria. For the recent update of the RECIST criteria to version 1.1, a large retrospective database of target lesion measurements was developed to test the impact of modifications to the criteria [84]. Meta-data on 18,000 potential target lesions were obtained from 6512 patients in 16 metastatic cancer clinical trials. The database was used to evaluate the impact of changes to RECIST on the classification of patient response to treatment [85]. The RECIST 1.1 criterion was thus validated only by comparing it to the previous standard approach and not by evaluating its correlation with survival endpoints. 

The ongoing multicenter I-SPY II (investigation of serial studies to predict your therapeutic response with imaging and molecular analysis 2) neoadjuvant breast cancer clinical trial [86] is attempting to address some of these issues by evaluating if breast MRI response predicts treatment outcomes. The NCI's cancer Biomedical Informatics Grid (caBIG) (https://cabig.nci.nih.gov/) program is supporting the I-SPY trial through integration and analysis of diverse data types including clinical, MRI imaging, and tumor biomarkers throughout the breast cancer treatment cycle. The integrated platform is designed to enable correlation of molecular data with MRI patterns to identify surrogate markers for early treatment response.

Despite these recent advances in the development of large databases to conduct research on response criteria, several limitations remain. First, these data repositories currently do not use data standards for sharing imaging metadata across institutions so as to enable the collection of large imaging data sets for research. Similarly, there is a lack of publicly available data sets containing baseline and follow-up imaging studies and image annotations, along with the corresponding diagnoses, therapies, and clinical outcomes. A publicly available database containing data from multiple institutions could be used to evaluate new image processing algorithms and response criteria.

### 6.3. Biomedical Informatics Systems to Support Response Assessment

Several biomedical informatics systems have been developed that could support the application and development of response criteria. These include systems to support (1) the creation of image repositories, (2) the acquisition, transfer and storage of image meta-data, (3) the automated interpretation of response data, and (4) the visualization of image meta-data for treatment decision making.

Several systems have been developed to support the creation of image repositories for clinical research. The National Biomedical Imaging Archive (NBIA) (https://cabig.nci.nih.gov/tools/NCIA) provides web-based access to deidentified DICOM images, markups, and annotations using role-based security. Publically available image collections available though the NBIA include the I-SPY trial and the Reference Image Database to Evaluate Therapy Response (RIDER) (https://wiki.nci.nih.gov/display/CIP/RIDER) which includes serial DCE-MRI studies of the breast. These publically available image data sets are available *via* the NCI hosted NBIA, but institutions can adapt the open source NBIA software for data storage by setting up a local instance of NBIA at the institution. 

The American College of Radiology Imaging Network (ACRIN) (http://www.acrin.org/) has developed the TRIAD (https://triad.acr.org) system to support the management of large image repositories for clinical research. The TRIAD web client allows users to search, download, and view available DICOM image series, while the graphical user interface client provides advanced functions for DICOM series routing, image processing, and annotation layer management. TRIAD is used to manage the image data for all of ACRIN's clinical trials but remains proprietary software.

In addition to advances in the management of clinical trial image data, several more recent advances have emerged in the management of image meta-data. Image meta-data refers to data that describe the content of images. Another initiative of the caBIG program was the development of the image Physician Annotation Device (iPad) [87]. iPad is a plugin to the popular open source OsiriX [88] image viewing application and implements the Annotation and Image Markup (AIM) standard [89]. AIM provides the data structure for storing the key semantic lesion information. “Semantics” refers to meaning in images including image metadata pertinent to quantitative criteria of disease, such as, lesion identification, location, size measurements, method of measurement, and other quantitative features. AIM utilizes the RadLex [90] controlled terminology for describing the contents of medical images, and provides a standard information model for semantic annotations. Information about image annotations is recorded in AIM as XML compliant with the AIM schema, enabling the consistent representation, storage, and transfer of the semantic meaning of imaging features. 

The use of iPad has been recently evaluated for tumor lesion tracking and semantic annotation of image meta-data to automatically populate lesion flow sheets for several cancer clinical trials [91]. The use of a semantic image annotation tool to directly populate lesion flow sheets provides several advantages. First, the use of an information model and structured terminology to encode cancer lesion image findings provides a consistent representation for storage and sharing of image meta-data needed for cancer clinical trials. The information model also provides a foundation to enable reasoning with and querying over image meta-data for response assessment. Image annotation disambiguates lesion identifiers by linking them directly to the source image and image mark-up. Image annotation tools also directly generate meta-data about image mark-up such as, length calculations eliminating possible transcription errors for length measurements.

Automated response assessment methods have also been developed to calculate RECIST variables such as, sum of diameters and the percent change from baseline. For example, a semantic reasoning method for response assessment utilizing AIM data was recently developed [92]. The method evaluates both the qualitative and quantitative features of tumor lesion annotations to calculate and classify treatment response according to the RECIST criteria. A vendor solution developed by MEDIAN technologies (http://www.mediantechnologies.com/) includes systems to measure and track cancer lesions and calculate quantitative RECIST metrics. Use of such automated approaches could improve the consistency and ease with which response criteria are applied in clinical research and routine care.

The abstraction and visualization of cancer lesion meta-data have also been demonstrated and integrated into the clinical work flow of treating medical oncologists [93]. Such visualizations enable rapid and accurate assessments of the image-based response data for use in treatment decision-making. Additional work however, is needed to incorporate image and response meta-data into the electronic health record and to integrate them with other clinical data such as treatments administered and laboratory response biomarkers.

### 6.4. Research Opportunities Bioinformatics Systems

**Figure 4:**
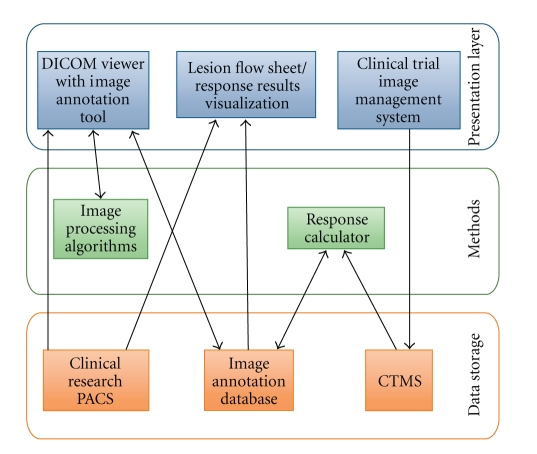
Biomedical informatics system architecture to support cancer treatment response assessment. Integrated systems are needed to support end-to-end management of response assessment data including data acquisition, analysis, and visualization. Data acquisition includes systems to acquire and store clinical images for research as well as systems to create image annotation data whether manual image annotation or automated image processing algorithms. Response calculation methods also need to be integrated with structured image annotation databases to perform data analysis to generate response interpretations. The images, image annotations, and response interpretations must be integrated with Clinical Trial Management Systems (CTMS) to enable visualization of response interpretations for treatment decision-making and auditing of clinical trial image response data.

We have described above several existing and emerging informatics systems that manage various aspects of image-based cancer treatment response assessment. However, several biomedical informatics research challenges remain to be solved to support application and development of response criteria for breast cancer. One of the greatest challenges remains integration of these various systems to support end-to-end data acquisition, interpretation and visualization for clinical trial data management and treatment decision support. Figure 4 shows an informatics architecture integrating multiple data acquisition and visualization systems with their respective data storage systems. Automated response calculators also need to be integrated into systems to support more consistent application of response criteria.

Research systems are also needed to better support the development of response criteria for novel imaging techniques such as DW-MRI and DCE-MRI of the breast. Large de-identified data sets are needed of image and image meta-data linked to patient treatments and clinical outcomes. While the iSPY trial presents an interesting pilot of such a system, larger data sets will likely be required to validate new response assessment modalities and criteria.

## 7. Breast MRI at 3T

For most of the past 20 years, human body imaging has been performed mainly at 1.5T. In recent years, clinical scanners operating at the higher field of 3T have become routinely available for clinical studies. To date, 1.5T is still the main field for studies of the breast.

### 7.1. Motivation for Higher Field Imaging

All magnetic resonance imaging techniques are constrained by a compromise between temporal and spatial resolution and signal-to-noise ratio (SNR). The fundamental motivations for high-field breast imaging are (1) a higher SNR, which provides gains that can then be kept or traded for higher spatial and/or temporal resolution, and (2) spectral spreading, which improves the resolution of magnetic resonance spectroscopy (discussed below in Section 7.3) and can improve fat suppression. These improvements can, potentially, lead to shortened acquisition times or increasing sensitivity and specificity for DW-MRI and DCE-MRI and, ultimately, better patient care. Unfortunately, direct comparison studies between 1.5 and 3T are difficult to execute due to the typically short pre-operative window in breast cancer and the requirement for a patient to make multiple trips to an imaging site because separate studies must be performed on separate days to eliminate image contamination by previous contrast material [94]. Thus, only a few studies have performed such a direct comparison, but they suggest that 3T is at least equivalent, and often better, than 1.5T.

Elsamaloty et al. [95] retrospectively examined sensitivity, specificity, and predictive values of breast MRI in 434 women and found MRI at 3T to be more sensitive than mammography and sonography (100% versus 81.8% and 86.4%, resp.) in detection of breast cancer and characterization of lesions smaller than 4 mm. They also found that 3T MRI had a higher sensitivity than lower-field MRI (100% versus 71%–94.4% reported for low-field studies) with no significant difference in specificity. Schmidt et al. [96] compared FDG-PET-CT to whole-body MRI at 1.5T and 3T and found that whole-body MRI using either field strength gave excellent sensitivity (93%) in detecting distant metastatic disease in breast cancer patients, but was less accurate at identifying involved lymph nodes (only 16 of 21 identified). Overall, they found “comparably good performance on both scanners with only slightly increased artifacts at 3T, without significantly restraining influence on image quality" and a shorter scan time at 3T (43 versus 51 min). An interesting example of a 3T breast protocol was offered by Pinker et al. [97]. Noting the conflicting demands of high temporal resolution imaging (required for quantitative DCE analysis) and high spatial resolution (required for clinical reading), the authors designed an imaging approach that alternates between a *T*_1_-weighted volume-interpolated-breathhold-examination (VIBE) sequence, for high temporal resolution data, and a *T*_1_-weighted fast-low-angle-shot (FLASH) sequence with fat suppression for morphologic analysis.

A final 3T versus 1.5T study we will discuss compared contrast-enhanced imaging of the breast in the same patient set at the two field strengths [98]. The goal of the study was to prospectively compare the the image quality and diagnostic performance of the data obtained at 3T and 1.5T. Image quality was (visually) scored from one to five, with one being nondiagnostic and five being excellent. Differential diagnosis was assessed by the receiver operator characteristic (ROC) analysis. Overall image quality was judged to be higher at 3T; 3T received an average score of 4.6 ± 0.5, while 1.5T received an average score of 4.2 ± 0.5 (*P* < .01). This difference in image quality translated into an improvement in diagnostic performance; an ROC score of 0.98 at 3T compared to. 0.84 at 1.5T (*P* < .05). The authors were careful to state that this result should not be read as an indication that contrast-enhanced breast MRI at 3T is more sensitive or specific than at 1.5T, but they did conclude that contrast-enhanced MRI at 3T is beginning to be ready for routine clinical use.

### 7.2. Challenges for Breast Imaging at 3T

Kuhl reviewed several issues involved with moving clinical breast imaging to 3T and stressed the need for maximum SNR [94]. Soher et al. provide an excellent review of relevant physics involved in going from 1.5 to 3T [99]. Major barriers to 3T imaging of the breast include increased ***B***_0_ and ***B***_1_ inhomogeneity (due to shorter RF wavelengths), higher specific absorption rate (SAR), changes in relaxation rate (lengthening *T*_1_ and shortening *T_2_*, which can adversely affect tissue contrast) changes, chemical shift artifacts, susceptibility gradients, and medical device safety. Some of the implications of these changes are described in more detail below.

As ***B***_0_ increases, the precession frequencies of nuclear magnets increase, in turn reducing the efficiency of spin-lattice relaxation processes and a longer persistence of the longitudinal magnetization. This translates to an increased *T*_1_ relaxation time at 3T. In order to preserve *T*_1_ contrast, a longer *T*_1_ would require longer repetition time (*TR*) and/or longer inversion delay values which may lead to lower temporal resolution and/or increased scan times. *T*_1_ values for both adipose and fibroglandular tissue have been shown to increase by approximately 20% in going from 1.5T to 3T [100]. This has a proportional effect on the inversion times used for fat suppression and provides a good example of sequence parameters that must change in going to 3T. Additionally, since the relaxivity of clinically accepted contrast agents decreases with field strength, dosing for DCE-MRI may need to be modified from the 1.5T values to achieve comparable contrast at 3T [101].

SAR scales with the square of the field strength, so all other parameters being equal, the SAR level at 3T is expected to be four times that at 1.5T. Increased SAR may require modification of pulse sequences, such as smaller flip angles, longer echo times, or more data (i.e., *k*-space) lines acquired per excitation, to reduce RF power deposition to 1.5T levels, thus compromising some of the SNR and temporal resolution gains available at 3T.

Increased ***B***_1_ inhomogeneity due to dielectric effects can cause spatial variations in SNR. The sensitivity of the overall scan is limited by the lowest sensitivity of any voxel in the volume so the overall scan sensitivity suffers when there are significant areas of low ***B***_1_. Azlan et al. have quantified ***B***_1_ inhomogeneity across the breast in normal volunteers at 3T [102]. They find a median 40% reduction of the expected ***B***_1_ in the right breast on *T*_1_-weighted spoiled gradient echo images.

### 7.3. Research Opportunities for Breast Imaging at 3T

Maintaining or improving image characteristics such as, contrast, resolution, and SNR at 3T will require not only modifications to conventional sequences but also improved hardware designs. Larger multichannel coil arrays will help maintain existing scan times with increased parallel acceleration factors while maintaining comparable SNR [103].

The stronger background magnetic field produces a wider separation of fat and water frequencies, though this improvement in spectral resolution is somewhat balanced by increased susceptibility effects that broaden the lines. Assuming that the line broadening does not completely nullify the wider separation of the spectral lines, fat suppression should be more complete at 3T. This gain, plus the possibility of increased time resolution, could improve fat-suppressed DW-MRI (important for limiting artifacts) as well as DCE-MRI studies [104]. However, ***B***_0_ and ***B***_1_ inhomogeneity make successful fat suppression difficult in practice. MR spectroscopy (MRS) is also hampered by the limited SNR available at 1.5T and could theoretically benefit from the increased SNR at 3T [105]. Many groups have employed MRS for measuring the response of tumors to treatment and the interested reader is referred to the excellent recent reviews in references [106, 107]. In the context of breast cancer, the MRS-visible molecule that has generated the most interest is choline. Since choline containing compounds are implicated in synthesis and metabolism of cell membranes, the standard interpretation of increases in total choline signal is increased cell turnover. However, at lower fields measuring this signal can be quite difficult and time consuming which can be a burden on the patient. It has been established that the sensitivity of proton MRS scales roughly linearly with field strength [108, 109]. Also, the increased spectral spreading with higher field strength leads to the separation of previously overlapping spectral peaks and ultimately the ability to resolve and quantify more compounds. Thus, the use of higher magnetic fields offers the opportunity for more precise and shorter MRS studies.

Finally, medical devices or implants deemed MR compatible at 1.5T may not be at 3T, or they may more markedly degrade the image quality as field strength increases [105]. However, Peters et al. concluded that MRI-guided large-core needle breast biopsy is safe and effective at 3T, despite fears over increased susceptibility artifacts from the biopsy needle [110]. More studies of device safety and effects on image quality must be done for the range of devices currently approved for 1.5T use.

## 8. Summary

In this paper we have highlighted several areas of quantitative magnetic resonance imaging that will have to be advanced in order to report with high sensitivity and specificity on the response of breast tumors to treatment. While the current state of the art in quantitative breast MRI is not quite ready for clinical acceptance, we believe that future developments in each of these areas will catalyze the use of more advanced MR techniques for breast cancer imaging. In fact, there are a number of organizations designed to assist in these efforts by standardizing various acquisition and analysis protocol; these include QIBA (Quantitative Imaging Biomarkers Alliance) and UPICT (Uniform Protocols for Imaging in Clinical Trials). There is also a recent NCI initiative to encourage the incorporation of emerging quantitative imaging in clinical trials (and, therefore, larger controlled patient populations) through the Quantitative Imaging Network (QIN).
